# Intergenerational Educational Inequality and Its Transmission in China’s Elite Universities

**DOI:** 10.3389/fpsyg.2022.813620

**Published:** 2022-03-07

**Authors:** Jianwen Wei, Shuanglong Li, Yang Han, Wangqian Fu

**Affiliations:** ^1^School of Sociology, Beijing Normal University, Beijing, China; ^2^Department of Sociology, School of Public Administration, Guangzhou University, Guangzhou, China; ^3^School of Public Health and Primary Care, The Chinese University of Hong Kong, Shatin, Hong Kong SAR, China; ^4^Faculty of Education, Beijing Normal University, Beijing, China

**Keywords:** family background, admission methods, academic performance, China, elite universities

## Abstract

China is experiencing high social inequality accompanying influential education reforms. The Independent Freshmen Admission (IFA) policy was one of the multiple strategies in higher education reforms in China against the social context of high social inequality and the expansion of higher education. By comparing students admitted through IFA with those admitted by the National College Entrance Examination (NCEE), we examined how family advantages contributed to higher education inequality in terms of educational opportunity, process, and results. Using data from an elite university in Beijing, we found that: (1) Family advantages improved a student’s likelihood of being admitted through IFA, exhibiting opportunity inequality. (2) No significant difference in academic grades existed between the students admitted through IFA and NCEE. In comprehensive quality, however, those recruited through IFA performed significantly better than those admitted through NCEE. (3) Family social capital not only increased the likelihood of students being admitted through IFA but also, through direct and indirect effects, increased their comprehensive quality performance in terms of receiving student association and social practice awards.

## Introduction

Education plays an essential role in modern society as it is a channel to achieve social mobility and socioeconomic status. However, it is also a tool for reproducing social inequality as family resources are important potential advantages for children’s educational opportunities and achievements (Blau and Otis, 1967; Wu, 2010). With the expansion of education, some scholars argued that family advantage contributed less to education inequality (Treiman, 1970), while other studies found that family background remained a significant effect on educational opportunities, despite the expansion of education (Shavit and Blossfeld, 1993; Buchmann and Hannum, 2001).

The effect of family background on higher education inequality should be discussed in the context of specific systems and backgrounds. China is a unique setting to study how social inequality contributed to education reproduction as it is experiencing a high social inequality accompanying significant reforms in the higher education system. On the one hand, China is experiencing high-income inequality measured by the Gini coefficient. A Gini coefficient is ranged from 0 to 1, with a higher score meaning higher income inequality. The Gini coefficient in China increased from 0.30 approximately in 1980 to 0.467 in 2017, indicating that a huge gap existed between the rich and the poor in the society (Xie and Xiang, 2014; Household Survey Office of National Bureau of Statistics, 2017); on the other hand, China has experienced huge changes in the education system since 1949—from the radical egalitarianism of the Cultural Revolution to the resumption of the National College Entrance Examination (NCEE). The influence of family background on educational inequality has been significantly different across different periods (Zhou et al., 1998; Li, 2010; Wu, 2010, 2011; Wu, 2013a,b; Yeung, 2013; Liu, 2014).

At the beginning of reform (i.e., the founding of the PRC in 1949), higher education was characterized by intense class struggle in terms of “equality within the class,” and political upbringing within the family was accordingly the primary cause for differentiated educational opportunities (Ying and Liu, 2015). Influenced by the dramatic increase in social stratification and differentiation since 1992, the educational system was stricken by marketization, revealing the effect of family resources, and the generational mechanism of educational inequality was further transformed into dual modes of resource conversion and cultural reproduction (Li, 2006). In the twenty-first century, higher education in China has undergone several fundamental changes. The expansion of higher education has significantly affected inequality in educational opportunities (Li, 2010; Ye and Ding, 2015). Aiming to solve the disadvantages of the traditional college enrollment system based on test scores, especially the questioning and criticism of the exam-oriented college entrance (i.e., NCEE), the Ministry of Education has also begun a large-scale reform of the college enrollment and examination system. One of the crucial strategies is Independent Freshmen Admission (IFA).

Independent Freshmen Admission is a comprehensive admission process that has challenged traditional admissions that are solely based on test scores, as it admits students through multifaceted assessment approaches such as individual applications, qualification evaluations, written examinations, and group interviews (Liu et al., 2014). The IFA of colleges and universities has become an important measure to change the disadvantages of the system of “one exam determines one’s life” and reflected the innovation and exploration of education and the requirements of quality education (Hu, 2020). IFA is only limited to a small number of elite universities, combining the independent admission program in selected universities with the NCEE (Wu et al., 2019). Students who have passed the IFA examination (including written test and interview) can enjoy special preferential treatment such as lower scores of the unified NCEE or preferential choice of major in enrollment (Wu et al., 2019). In addition to providing an alternative path toward higher education nationwide, the purpose of IFA is to recruit outstanding students with academic specialization and innovative potential who could be missed in the NCEE (Ministry of Education, 2012; Wu et al., 2019). The problem of inequality is equally present in the IFA process (Bao, 2012; Ma and Bu, 2019). The new educational opportunities offered by the IFA system may benefit only the students from higher socioeconomic status (SES) families, and the most privileged educational resources would be continuously used by advantaged groups (DiPrete and Gregory, 2006; Liu et al., 2014; Li, 2016; Qian and Yang, 2019).

Although previous studies have examined the effect of higher education expansion on educational inequality, few have studied how the IFA, the new admission method among several strategies in the education reform and expansion, affects higher education inequality in China. Thus, this study filled the gap by answering the research questions: (1) whether family background affects IFA and the subsequent academic performance of students, and (2) whether it contributes to retaining the advantages of students from privileged backgrounds in achieving educational success.

## Literature Review and Research Hypotheses

Education is crucial in attaining higher social status and achieving upward social mobility in modern societies. Existing literature has demonstrated that people in prominent positions are those who have been educated (Treiman and Yip, 1989; Muller and Shavit, 1998); accordingly, education is positioned at the core of studies on social stratification, and educational attainment has become a vital subfield in the study of intergenerational mobility (Shavit and Blossfeld, 1993; Hannum and Yu, 1994; Deng and Treiman, 1997; Li, 2006; Wu, 2011).

### Family Background and Educational Opportunity

Family background has a vital function in educational attainment, and a privileged family social status is advantageous for acquiring educational opportunities. From the microscopic perspective of Blau and Otis (1967) status attainment model, family social resources are explained as potent factors in children’s educational attainment. Studies have uncovered, despite the expansion of education, family background has continued to exert a significant influence on educational opportunities, regardless of whether a child lives in a developed or developing country (Treiman and Yip, 1989; Shavit and Blossfeld, 1993; Buchmann and Hannum, 2001; Wu, 2011). Research in China has similarly shown that educational inequality has persisted alongside educational expansion, suggesting that the effect of the family background remains significant (Li, 2010; Ye and Ding, 2015).

Previous sociological studies have focused on several institutional contexts when discussing the family background and educational inequality: first, institutional transformation (i.e., the marketization revolution) (Zhou et al., 1998; Li, 2006; Wu, 2010; Li, 2014); second, educational expansion since the expansion of higher education recruitment in 1998 (Li, 2010; Wu, 2013a); and third, the establishment of the key secondary school and tracking systems (Wu, 2013b; Tam and Jin, 2015). Since 2003, implementing an independent recruitment system (i.e., IFA) has provided an alternative institutional context for examining the relationship between family background and educational inequality.

In the 21st century, reform in the Chinese higher education system has given birth to the IFA. If the expansion of higher education recruitment in 1998 can represent an educational expansion in quantity, then the IFA can be regarded as increasing the pathways for educational attainment (Liu et al., 2014). From a theoretical point of view, “maximally maintained inequality (MMI, hereafter)” (Raftery and Michael, 1993) provides a noteworthy explanation for the relationship between educational expansion and inequality. According to MMI, when educational opportunities increase, families of privileged social status continue to control a large share of the educational resources, and only when the educational attainment of these privileged individuals is maximized can the benefit of these increasing educational opportunities reach individuals of lower social status. In addition, family cultural capital exerts a significant effect on the educational attainment of a child (Bourdieu, 1974). Families with higher cultural capital place greater emphasis on education and are thus willing to pay higher costs; parents in such families may also have higher capabilities to assist in their children’s learning. Influenced by their family background, these children have higher cultural capital, place greater emphasis on education, and accordingly achieve higher educational performance (De Graaf et al., 2000; Sullivan, 2001; Li, 2006). The social capital of a family is another crucial factor in educational opportunities. Social capital is the sum of actual or potential resources attainable from institutionalized social networks; the unequal distribution of social capital thereby results in disparate educational attainment among children of differing family backgrounds (Bourdieu, 1986).

Through more subjective and flexible methods such as initial qualification evaluations, paper examinations, and groups interviews, the range of standardized assessments in the IFA is far greater than the knowledge that students acquire in school, rendering it less objective than standardized examinations and consequently beneficial to students with privileged family backgrounds. Students with higher social and cultural capital are more likely to be admitted through IFA than those with lower social and cultural capital, meaning that this system contributes to educational inequality. The following research hypothesis is thus proposed:

*Hypothesis 1: University students from more privileged family backgrounds are more likely to be admitted into higher education through IFA than those who are less privileged.*

### Admission Methods and Academic Performance

The purpose of the IFA system is to “select innovative talents, cultivate specializations, and actively explore a new system that, with standardized examination as its base, integrates diversified examinations, versatile admission selections, and independent recruitment to effectively select outstanding and innovative talents through academic autonomy, comprehensive government instruction, and service” (Ministry of Education, 2003). In the recent decade, the Chinese Ministry of Education has further defined IFA as intended for students with “academic specialization” and “innovative potential” (Ministry of Education, 2011, 2013). Through step-by-step assessment procedures (i.e., qualification evaluations, paper examinations, and interviews), IFA features more comprehensive and practical assessments to identify the capabilities of an applicant among the massive number of applications; under such institutional requirements, students who can stand out from the rest are therefore more competent. This study thus proposes that disparities exist between students admitted through the two different systems. The second research hypothesis is, accordingly:

*Hypothesis 2: Academic achievement among university students admitted through IFA is higher than university students admitted through NCEE.*

### The “Dual Pathway” Effect of Family Background on Academic Performance

The literature review revealed that family background significantly affects university admissions, and disparities may similarly exist between individuals who entered universities through different admission methods: students admitted through IFA achieve higher academic performance than students who took the standardized NCEE. Furthermore, the effects of family background on academic performance can be divided into direct and indirect influences. Two types of admission methods mediate the effect of family background on academic performance. Through IFA, the privilege of family background is transformed into the advantage of academic achievement, further widening disparities in the social hierarchy. The social capital of family background may affect academic performance among university students primarily because the networking resources generated by family social capital provide these university students with greater educational opportunities that foster their comprehensive quality in various aspects.

The third hypothesis of this study is therefore proposed as follows:

*Hypothesis 3a: Holding other factors constant, family background displays a significant and direct influence on academic performance.*
*Hypothesis 3b: Family background affects academic achievement indirectly by influencing the admission method.*

## Data and Methods

### Data

Data were collected from 2011 to 2014 among undergraduates in an elite university in Beijing. According to Population Census data in 2010 (National Bureau of Statistics of China, 2012), which is one year before our first wave survey, around 118 million people in China had completed tertiary education (including college, undergraduate, and postgraduate), accounting for 8.64% of the total population in 2010 in China. The proportion of people getting a bachelor’s degree in elite universities is even much lower than the 8.64%, which means the opportunity of getting an education in our selected university is rare. A random sampling method was adopted to select undergraduates of all years and majors. First, a list of all undergraduates was obtained from the student affairs office, and the research participants were retrieved through a random sampling procedure using SPSS software. Subsequently, the student affairs office invited the selected students through the school counselors in each school/department and asked them to complete a questionnaire in different classrooms. Supervisor were employed to examine the quality of the answers and collect the questionnaires on site. A total of 5422 valid questionnaires were collected over the 4-year survey. The numbers of valid questionnaires acquired each year were as follows: 2,014 in 2011; 1,298 in 2012; 1,102 in 2013; and 1,008 in 2014.

### Variables

**FIGURE 1:**
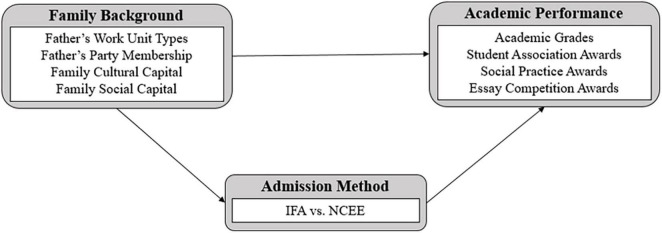
Analysis framework.

Key variables in this study are shown in the analysis framework (Figure 1). The first step of the study involved examining the influence of family background on the admission method. The admission method was adopted as a dependent variable and subsequently processed into a dummy variable: admission through IFA was coded as 1, and admission through the standardized NCEE was coded as 0.

The key independent variables were family background, measured by father’s work unit, father’s membership in the Chinese Communist Party (CCP), family cultural capital, and family social capital. Father’s work unit was categorized into three categories: 1 = private organizations; 2 = state-owned enterprises; 3 = Party (i.e., CCP) or government organizations. Father’s Party membership is a dummy variable, with non-party members as the reference group.

Family cultural capital was evaluated using average parental educational attainment, with a maximum measure of 12 years: average parental educational attainment below 12 years was labeled “1 = low family cultural capital”; average parental educational attainment above 12 years but below 16 years was labeled “2 = intermediate family cultural capital”; average parental educational attainment greater than or equal to 16 years was labeled as “3 = high family cultural capital.”

The position generator technique in the “Chinese Lunar New Year greeting network” (Bian and Li, 2002) was adopted to measure family social capital. We assigned a power index to each occupation within one’s network (Wei and Zhao, 2011) and then summed it. The sum of family social capital was coded as follows: the first 25% of the lowest power index was coded as 1 = “low family social capital”; a power index ranging between 25 and 75% was denoted as 2 = “intermediate family social capital”; and power index higher than 75% was coded as 3 = “high family social capital.”

The second step of our analysis is to examine the effects of family background and the admission method using both of these as the core independent variables. Academic performance was employed as the dependent variable and was classified primarily into academic grades and comprehensive quality. Additionally, academic grades were measured using the overall class ranking from the previous semester (1 = low; 2 = low-intermediate; 3 = intermediate; 4 = upper-intermediate; 5 = superior); comprehensive quality included whether the participants received awards in student associations, social practices, and essay competitions (1 = awarded; 0 = not awarded).

The third step of the analysis was investigating the direct influence of family background on academic performance and the indirect influence generated through the admission method, with variables measured the same as in Step 1 and 2.

The control variables included in this study were as follows: gender (1 = male; 0 = female), enrollment age (continuous variable), area of study in high school (1 = humanities; 0 = sciences), registered household residence (*hukou*) before admission (1 = urban; 0 = rural), family residence (1 = city; 2 = county; 3 = town or village), ethnicity (1 = Han; 0 = minority ethnic groups), and rank/type of secondary school (1 = national key level; 2 = provincial level; 3 = city level and below). In addition, the frequency of self-study was included in the analysis on academic achievement in Step 2 to control for the level of individual effort (1 = never; 2 = rarely; 3 = occasionally; 4 = sometimes; 5 = often).

## Methods

The statistical analyses proceed in three steps. In the initial stage, a binary logistic regression model was employed, with the dependent variables as a binary variable. In the second step, ordinal logistic regression and binary logistic regression models were applied given that academic grade (i.e., overall class ranking) was an ordinal variable, and awards in student associations, social practices, and essay competitions were binary variables. Finally, the decomposition method developed by Buis (2010) was used to analyze the direct and indirect effect of family background on academic performance since the decomposition was based on logistic regression models.

## Results

### Sample Characteristics

Table 1 shows the sample characteristics. In our survey, 63.04% of respondents were male. The mean enrollment age was 18.28 (SD = 0.793). Among the respondents, 69.76% studied science in high school, 82.48% held an urban *hukou* before being admitted to the university, and 60.48% came from cities.

**TABLE 1:** Statistical descriptions of the key variables used.

	Whole Sample	IFA	NCEE	N
**Controls**				
Gender (%)				
Male	36.96	41.98	35.98	5336
Female	63.04	58.02	64.02	
Enrollment age [Mean (SD)]	18.28 (0.793)	18.20 (0.742)	18.29 (0.802)	5214
Area (%)				
Science	69.76	60.05	71.63	5159
Humanities	30.24	39.95	28.37	
Hukou (%)				
Rural	17.52	7.84	19.40	5178
Urban	82.48	92.16	80.60	
Family Residence (%)				
City	60.48	78.82	56.92	
County	23.30	15.98	24.71	5207
Town or village	16.23	5.21	18.36	
Ethnicity (%)				
Minority	13.66	9.10	14.55	5211
Han	86.34	90.90	85.45	
Rank/Type of Secondary School (%)				
National Key Level	11.00	12.69	10.68	
Provincial level	47.31	54.21	45.97	5198
City level and below	41.69	33.10	43.35	
**Family background**				
Father’s Work Unit Types (%)				
Private organizations	31.26	22.43	33.07	4702
State-owned enterprises	44.13	42.73	44.42	
Party or government organizations	24.61	34.84	22.52	
Father’s Party Membership [Mean (SD)]	0.50 (0.070)	0.72 (0.016)	0.49 (0.008)	4925
Family Cultural Capital (%)				
Low	33.24	13.91	37.03	4828
Intermediate	34.86	33.50	35.13	
High	31.9	52.59	27.84	
Family Social Capital (%)				
Low	24.88	15.25	26.74	5214
Intermediate	49.60	49.17	49.68	
High	25.53	35.58	23.58	
**Academic performance**				
Overall Class Ranking (%)				
Low	4.82	5.10	4.77	3567
Low-intermediate	18.73	19.28	18.63	
Intermediate	29.55	33.08	28.93	
Upper-intermediate	31.37	26.84	32.16	
Superior	15.53	15.69	15.50	
Student association awards [Mean (SD)]	0.18 (0.007)	0.26 (0.019)	0.17 (0.007)	3411
Social practice awards [Mean (SD)]	0.28 (0.008)	0.35 (0.021)	0.27 (0.008)	3427
Essay competition awards [Mean (SD)]	0.07 (0.004)	0.11 (0.015)	0.06 (0.005)	3194

*Standard deviations in parentheses.*

### Influence of Family Background on the Admission Method

Table 2 displays the results of the logistic regression model. Model 1 is the baseline model, which only included the controlled variables that possibly influenced the admission method. The variable of family social status was added in Model 2. According to Model 2, father’s work unit types and Party membership significantly influenced the admission method (*p* < 0.001). When other variables were controlled for, the odds of IFA among participants whose fathers worked in Party or government organizations increased by 69% (e^0.526^–1) compared to those whose fathers worked in private organizations; students whose fathers were party members had a 74% (e^0.553^–1) higher odds of being admitted through IFA compared to those whose fathers were non-party members.

**TABLE 2:** The effects of family background on admission method.

	Model 1	Model 2	Model 3	Model 4
**Independent variables**				
Father’s Work Unit Types (Ref.: Private organizations)				
State-owned enterprises		0.182	0.0920	0.0753
		(0.116)	(0.119)	(0.119)
Party or government organizations		0.526***	0.409**	0.376**
		(0.125)	(0.128)	(0.129)
Father’s Party Membership (Ref.: No)		0.553***	0.355***	0.334**
		(0.0988)	(0.103)	(0.103)
Cultural Capital (Ref.: Low)				
Intermediate			0.544***	0.491***
			(0.148)	(0.149)
High			1.085***	1.018***
			(0.150)	(0.151)
Social Capital (Ref.: low)				
Intermediate				0.302*
				(0.123)
High				0.511***
				(0.135)
*N*	5099	4571	4454	4454
pseudo *R*^2^	0.069	0.085	0.101	0.105

Standard errors in parentheses; *p < 0.05, **p < 0.01, ***p < 0.001. We controlled gender, area of study, hukou before admission, family residence before admission, ethnicity, the rank of secondary school, and survey year in Model 1 to Model 4 (see Supplementary Table 2 for details).

In Model 3, family cultural capital was added. The results revealed that holding other factors constant, participants from family backgrounds with intermediate cultural capital displayed a 72% (e^0.544^–1) (*p* < 0.001) higher odds of being admitted *via* IFA compared to those from families with low cultural capital; moreover, participants from families with high cultural capital were 2.96 (e^1.085^) times more likely (*p* < 0.001) to be admitted through IFA than participants from families with low cultural capital. In both conditions, the effects of father’s work unit and Party membership remained significant (*p* < 0.001).

In Model 4, family social capital was employed based on Model 3. According to the results, family social capital had significant effects on the admission method (*p* < 0.05). When other variables were controlled for, participants with intermediate family social capital demonstrated a 35% (e^0.302^–1) (*p* < 0.05) higher odds of being admitted *via* IFA than did those with low family social capital; furthermore, participants with high family social capital manifested a 67% (e^0.511^–1) (*p* < 0.001) higher odds of being admitted *via* IFA than did those with low family social capital. In both models, the effects of the father’s work unit, Party membership, and family cultural capital remained positive and significant (*p* < 0.05).

### Influence of Family Background and the Admission Method on Academic Performance

Model 5 in Table 3 revealed that when other variables were controlled for, no significant difference was found in the relationship between family background or the admission method and overall class ranking (*p* > 0.05). Models 6, 7, and 8 were the results of binary logistic regression models. According to the results of Model 6, when other variables were controlled for, students with high family social capital achieved a 59% (e^0.461^–1) higher odds of achievement in student associations than did students with low family social capital (*p* < 0.01), and students who were admitted through IFA attained a 38% (e^0.319^–1) higher odds of obtaining student association award than did those who took the NCEE (*p* < 0.05).

**TABLE 3:** The effects of family background and admission method on academic performance.

	Mode 5	Mode 6	Mode 7	Mode 8
	Overall class ranking	Student association awards	Social practice awards	Essay competition awards
**Independent variables**				
Father’s Work Unit Types (Ref.: Private organizations)				
State-owned enterprises	–0.0101	0.198	0.145	–0.118
	(0.0908)	(0.132)	(0.113)	(0.156)
Party or government institutions	–0.0765	0.187	0.00187	–0.140
	(0.105)	(0.151)	(0.132)	(0.178)
Father’s Party Membership	0.119	–0.0220	0.0459	0.187
(Ref.: No)	(0.0807)	(0.116)	(0.100)	(0.137)
Cultural Capital (Ref.: Low)				
Intermediate	0.0191	–0.123	0.0545	–0.0539
	(0.0990)	(0.147)	(0.124)	(0.172)
High	–0.0578	0.0347	–0.0782	–0.0258
	(0.108)	(0.154)	(0.136)	(0.183)
Social Capital (Ref.: Low)				
Intermediate	0.0413	0.267	0.306**	0.108
	(0.0861)	(0.136)	(0.114)	(0.154)
High	–0.0418	0.461**	0.497***	0.152
	(0.103)	(0.153)	(0.132)	(0.178)
Admission Method	–0.0782	0.319*	0.329**	0.279
(Ref.: NCEE)	(0.0986)	(0.130)	(0.118)	(0.157)
*N*	3011	2960	2974	2796
pseudo *R*^2^	0.058	0.037	0.037	0.052

Standard errors in parentheses; *p < 0.05, **p < 0.01, ***p < 0.001. We controlled gender, area of study, hukou before admission, family residence before admission, ethnicity, the rank of secondary school, grade, frequency of self-study, and survey year in Model 5 to Model 8 (see Supplementary Table 2 for details).

The results of Model 7 displayed similarities to those of Model 6: when other variables were controlled for, students with high family social capital displayed a 64% (e^0.497^–1) higher odds of obtaining social practice award than did students with low family social capital (*p* < 0.001); students with intermediate family social capital displayed a 36% (e^0.306^–1) higher odds of than did those of low family social capital (*p* < 0.01); and students admitted through IFA manifested a 39% (e^0.329^–1) higher odds of obtaining social practice awards (*p* < 0.01). Model 8 displays the effects of family background and admission method on essay competition awards. The results showed no significant effect of family background and the admission method on essay competition award (*p* > 0.05).

### Direct and Indirect Effect of Family Background

Table 3 displays the findings of Models 6 and 7 on the significant influences of family social capital and the admission method on student association awards and social practice awards (*p* < 0.05); similarly, the significant influence of family social capital on admission method is demonstrated by Model 4 in Table 2. Based on these above-mentioned findings, this section shows the extent to which these influences were directly generated from family social capital, and to what extent they were indirectly influenced by the effect of family social capital on academic achievement through admission method.

In Table 4, the direct and indirect effects of family background on receiving awards were estimated using the decomposition method developed by Buis (2010). As shown in Model 9, when the distribution of admission methods is the same between students from family backgrounds with low and high social capital, the achievement odds of obtaining student association award among students with low family social capital can be enhanced by 5% (e^0.047^–1) as an indirect effect through admission method (*p* < 0.01); when admission method was controlled for, students with high family social capital attained a 73% (e^0.551^–1) higher odds of obtaining student association award than did those with low family social capital as a direct effect of family social capital (*p* < 0.001). Similar differences were observed between students from families with intermediate and low social capital and between students from families with high and intermediate social capital.

**TABLE 4:** The direct and indirect effects of family background on receiving awards.

	Mode 9	Mode 10
	Student association award	Social practice award
High/Low		
Total effects	0.598*** (0.123)	0.603*** (0.132)
Indirect effects	0.047** (0.018)	0.046** (0.018)
Direct effects	0.551*** (0.121)	0.557*** (0.137)
Intermediate/Low		
Total effects	0.312* (0.132)	0.352** (0.118)
Indirect effects	0.027* (0.017)	0.028* (0.012)
Direct effects	0.285* (0.132)	0.323** (0.122)
High/intermediate		
Total effects	0.286* (0.114)	0.252** (0.095)
Indirect effects	0.019* (0.010)	0.018* (0.008)
Direct effects	0.266* (0.113)	0.234* (0.095)
*N*	2960	2974

Bootstrap standard errors in parentheses; *p < 0.05, **p < 0.01, ***p < 0.001.

Model 10 showed the effect of family social capital on the social practice awards, displaying similar results to Model 9. If the distribution of admission methods is the same between families with low and high social capital, the odds of obtaining social practice awards among students from families with low social capital increased by 5% (e^0.046^–1) as an indirect effect through admission method (*p* < 0.01). When the admission method was controlled for, students from families with high social capital had a 75% (e^0.557^–1) higher odds of obtaining social practice award than did students from families with low social capital (*p* < 0.001). Similar results were identified between students from families with intermediate and low social capital and students from families with high and intermediate social capital.

Table 5 also displays the direct effect of family social capital and the indirect effect of family social capital through admission methods on student association and social practice awards. The main diagonal indicates the factual odds of obtaining each award for each group.

**TABLE 5:** Predicted proportions of receiving awards and counterfactual proportions (%).

	Student association awards			Social practice awards		
	Association			Association		
Distribution	Low	Intermediate	High	Low	Intermediate	High
Low	13.5	17.2	21.4	21.5	27.5	32.4
Intermediate	13.9	17.6	21.8	22.0	28.0	33.0
High	14.1	17.9	22.2	22.3	28.4	33.4

Each row indicates the direct effect of family social capital on obtaining awards when the admission method was controlled for. Each column indicates the indirect effect of family social capital on obtaining awards through the admission method; in other words, the effect of family social capital on obtaining awards may vary when the admission method distribution changes. For instance, 13.5% of the students with low family social capital earned student association awards. If the distribution of admission methods of the students with low family social capital did not change, and their family social capital was the same as that of the students with high family social capital, the proportion of obtaining student association awards among the students with low family social capital would increase from 13.5 to 21.4%. If the distribution of admission methods was the same between families with low and high social capital, the proportion of obtaining student association awards among the students with low family social capital would increase to 14.1%. Similar results were obtained regarding social practice awards.

## Discussion and Conclusion

Unequal access to higher education between students from different class backgrounds is a global issue. Many countries, including the United States and Brazil, have adopted affirmative action to improve educational equity (French, 2021). In 2012, the Brazilian government initiated the quota law requesting all public universities to allocate 50% of the admission vacancies to public high school graduates based on the criteria such as race and income. Although time has not quelled controversy over this policy (Turgeon and Habel, 2021), extant research suggests no significant difference in academic performance between quota and non-quota students, indicating that this policy is worthy of recognition (Vidigal, 2018; Pelegrini et al., 2022). In the United States, both SAT and ACT examination has undergone significant changes to boost enrollment of lower socioeconomic and racial/ethnic groups (Hurwitz et al., 2017). However, there are still substantial class differences in test prep for college entrance exams and private tutoring, which can significantly increase test scores and college admissions chances (Moore et al., 2018; Kim et al., 2021). Multi-dimensional and comprehensive admission indicators were initially used to ensure that students from the bottom of society have equal opportunities to enter college. Nonetheless, in practice, the ambiguity of admission criteria has led to most opportunities occupied by students from advantageous families (Rosinger et al., 2021). In addition, exam retakes could increase students’ chances of entering a college, but students from poor and minority backgrounds have lower retake rates than students from wealthier families (Goodman et al., 2020).

Since 1949, China’s elite higher education has undergone many changes, including the “silent revolution” (Liang et al., 2012), which has attracted much controversy and attention. In this study, an elite university in Beijing was used as a case to compare differences between the family backgrounds and academic performances of students admitted through IFA and NCEE. The influence of IFA on educational inequality was examined and controversies surrounding the silent revolution were discussed. The primary findings are as follows:

In terms of admission methods, university students with more privileged family backgrounds had a higher likelihood of being admitted into higher education through IFA, revealing significant inequality in educational opportunity. It is consistent with the existing studies (Qian and Yang, 2019; Wang, 2019; Wu et al., 2019). As a system design for selecting elite talents, IFA naturally tends to favor urban families with a considerable accumulation of cultural capital in the evaluation methods, assessment contents, and implementation procedures (Qian and Yang, 2019). This situation is evident when the following occurs: advantages in family social status, father’s party membership status, and the father’s employment in a party or government organization significantly increased a child’s likelihood of being admitted through IFA. It is worth noting that the fathers’ party status’ influence in China may be related to China’s political capital, providing high social status and income (Wang and Li, 2020). The high social status contributes to the family’s use of social networks to get information and connect with relevant people (Liu, 2018). In addition, advantages in family cultural and social capitals substantially increased a child’s likelihood of being admitted through IFA.

Students admitted through IFA and NCEE showed significant differences in comprehensive quality. No significant difference existed between students admitted through IFA and NCEE regarding academic grades measured by overall class ranking in our study. In terms of comprehensive quality, however, students admitted through IFA performed significantly better in award obtaining than those admitted through NCEE. That means students admitted through IFA comprehensively perform better when they enter universities (Guo, 2020). It may be because the students with advantaged family backgrounds have better ability in the independent recruitment process, such as speech, self-exploration, artistic talent, etc. (Liu, 2018). Also, the students selected by universities through IFA are not necessarily the most competent but the most suitable for the school’s talent cultivation model (Ma and Bu, 2019).

The influence of family social capital was significant in conveying educational inequality. Family social capital significantly affected both the student association awards and social practice awards of students admitted through independent recruitment. High family social capital increased the likelihood that a student can receive both types of awards. The effect mechanism of family social capital had a direct influence; the indirect benefits created through the admission method were non-significant.

Some limitations remain in this study. Firstly, these analyses were restricted to students of one elite university admitted in 2011 and 2014. Thus, it may not be suitable to generalize to the whole country. Secondly, the samples analyzed here were restricted to students who attend universities in Beijing only. Further work with a nationally representative sample is expected to reveal the whole picture. However, we focus on the mechanisms of educational inequality, that is, the underlying mechanisms by which different admissions approaches play a role. Given that the specific implementation policy of IFA has not changed in the past few years, the conclusion of our research will not be substantially affected by the timeliness of the data. Also, as the specific implementation policy of IFA is similar among each university, we speculate that the mechanisms could also be applicable to other universities in China. Thirdly, there is a lack of unified definition and evaluation of comprehensive quality among domestic universities in China, so our measurement of comprehensive quality may be biased. We hope future research can conduct a more comprehensive measurement and comparison of their performance and a more detailed tracking survey comparing their employment rate and postgraduate entrance examination rate after graduation.

The focus of the controversy over the IFA contradiction between difference and equality is the balance of talent selection and educational equity. It is also the key needed to be considered in the reform and optimization of college entrance examination policy (Qian and Yang, 2019). In order to make up for the unequal right to education in the independent enrollment policy, the comprehensive evaluation enrollment system and the special national plan were introduced accordingly in China. Safeguarding education equity requires diversified selection approaches rather than a simple “one examination system” model. Additionally, regional differences should be balanced. More attention and educational resources should be given to rural and educationally backward areas so that more students in disadvantaged areas can get more opportunities to attend higher education institutions. Since 2020, to improve the fairness of admission to Chinese elite universities, elite universities must allocate a certain percentage of vacancies to students from remote, poor, populated, and minority-inhabited areas. We expect more follow-up studies that provide timely analysis of the substantive consequences of the new policy.

## Data Availability Statement

The raw data supporting the conclusions of this article will be made available by the authors, without undue reservation.

## Ethics Statement

The studies involving human participants were reviewed and approved by the Central University of Finance and Economics, China. The patients/participants provided their written informed consent to participate in this study.

## Author Contributions

JW and SL designed the research. YH and WF analyzed the data. All authors wrote the manuscript, contributed to the article, and approved the submitted version.

## Conflict of Interest

The authors declare that the research was conducted in the absence of any commercial or financial relationships that could be construed as a potential conflict of interest.

## Publisher’s Note

All claims expressed in this article are solely those of the authors and do not necessarily represent those of their affiliated organizations, or those of the publisher, the editors and the reviewers. Any product that may be evaluated in this article, or claim that may be made by its manufacturer, is not guaranteed or endorsed by the publisher.
