# Supplementary material for: Intergenerational Educational Inequality and Its Transmission in China’s Elite Universities
Source: Front Psychol. 2022 Mar 7;13:813620. doi: 10.3389/fpsyg.2022.813620 (PMC8940217; doi:10.3389/fpsyg.2022.813620)
Supplement: Supplementary file 1 [file Data_Sheet_1.docx]

**Supplementary Material**

**Table S1. The Effects of Controls variables on Admission Method**

|  | **Model 1** | **Model 2** | **Model 3** | **Model 4** |
| --- | --- | --- | --- | --- |
| **Controls** |  |  |  |  |
| Gender (Ref.: Female) | 0.412^***^ | 0.410^***^ | 0.406^***^ | 0.414^***^ |
|  | (0.0816) | (0.0857) | (0.0877) | (0.0880) |
| Enrollment age | -0.160^**^ | -0.137^*^ | -0.117^*^ | -0.114^*^ |
|  | (0.0525) | (0.0555) | (0.0569) | (0.0570) |
| Area (Ref.: Science) | 0.629^***^ | 0.605^***^ | 0.648^***^ | 0.643^***^ |
|  | (0.0832) | (0.0878) | (0.0902) | (0.0904) |
| Hukou (Ref.: Rural) | 0.282 | 0.0174 | -0.199 | -0.215 |
|  | (0.174) | (0.190) | (0.199) | (0.199) |
| Family Residence (Ref.: City) | | | | |
|  |  |  |  |  |
| County | -0.673^***^ | -0.655^***^ | -0.412^***^ | -0.448^***^ |
|  | (0.106) | (0.110) | (0.115) | (0.115) |
| Town or village | -1.309^***^ | -1.257^***^ | -0.906^***^ | -0.886^***^ |
|  | (0.202) | (0.226) | (0.236) | (0.236) |
|  |  |  |  |  |
| Ethnicity (Ref.: Minority) | 0.542^***^ | 0.568^***^ | 0.632^***^ | 0.635^***^ |
|  | (0.135) | (0.144) | (0.147) | (0.148) |
|  |  |  |  |  |
| Rank/Type of Secondary School (Ref.: National Key Level) | | | | |
|  |  |  |  |  |
| Provincial level | 0.120 | 0.0878 | 0.151 | 0.142 |
|  | (0.125) | (0.129) | (0.133) | (0.134) |
|  |  |  |  |  |
| City level and below | -0.168 | -0.139 | -0.0940 | -0.0771 |
|  | (0.131) | (0.136) | (0.141) | (0.141) |
| Year (Ref.: 2011) |  |  |  |  |
|  |  |  |  |  |
| 2012 | 0.417^***^ | 0.402^***^ | 0.433^***^ | 0.448^***^ |
|  | (0.0982) | (0.102) | (0.105) | (0.105) |
| 2013 | -0.0574 | -0.0674 | -0.0618 | -0.143 |
|  | (0.111) | (0.120) | (0.123) | (0.125) |
| 2014 | -0.239^*^ | -0.243^+^ | -0.182 | -0.137 |
|  | (0.119) | (0.127) | (0.131) | (0.132) |
| Constant | 0.399 | -0.360 | -1.204 | -1.477 |
|  | (0.999) | (1.059) | (1.093) | (1.098) |
| *N* | 5099 | 4571 | 4454 | 4454 |
| pseudo *R*^2^ | 0.069 | 0.085 | 0.101 | 0.105 |

Standard errors in parentheses

^+^ *p* < 0.1, ^*^ *p* < 0.05, ^**^ *p* < 0.01, ^***^ *p* < 0.001

**Table S2. The Effects of Control Variables on Academic Performance**

|  | **Mode 5** | **Mode 6** | **Mode 7** | **Mode 8** |
| --- | --- | --- | --- | --- |
|  | **Overall Class Ranking** | **Student Association Award** | **Social Practice Award** | **Essay Competition Award** |
| **Controls** |  |  |  |  |
| Gender (Ref.: Female) | -0.891^***^ | 0.219^*^ | -0.118 | 0.000580 |
|  | (0.0726) | (0.102) | (0.0893) | (0.122) |
| Area (Ref.: Science) | -0.0437 | 0.0303 | -0.0888 | -0.0569 |
|  | (0.0734) | (0.107) | (0.0927) | (0.129) |
| Hukou (Ref.: Rural) | -0.102 | 0.0325 | -0.115 | -0.114 |
|  | (0.128) | (0.208) | (0.164) | (0.240) |
| Family Residence (Ref.: City) |  |  |  |  |
|  |  |  |  |  |
| County | -0.0961 | -0.404^**^ | 0.00999 | -0.223 |
|  | (0.0871) | (0.131) | (0.109) | (0.153) |
| Town or village | -0.294^*^ | -0.456^*^ | 0.221 | -0.427 |
|  | (0.138) | (0.224) | (0.174) | (0.260) |
|  |  |  |  |  |
| Ethnicity (Ref.: Minority) | 0.643^***^ | -0.0868 | 0.133 | 0.370 |
|  | (0.0989) | (0.145) | (0.129) | (0.194) |
| Rank/Type of Secondary School (Ref.: National Key Level) | | | | |
|  |  |  |  |  |
| Provincial level | -0.0372 | -0.125 | -0.276^*^ | -0.310 |
|  | (0.112) | (0.152) | (0.134) | (0.175) |
| City and below | -0.314^**^ | -0.169 | -0.371^**^ | -0.256 |
|  | (0.114) | (0.157) | (0.138) | (0.179) |
| Grade (Ref.: Sophomore) |  |  |  |  |
|  |  |  |  |  |
| Junior | 0.104 | 0.0467 | 0.0528 | 0.848^***^ |
|  | (0.0777) | (0.112) | (0.0944) | (0.145) |
| Senior | 0.262^**^ | -0.188 | -0.698^***^ | 0.774^***^ |
|  | (0.0870) | (0.129) | (0.116) | (0.157) |
| Frequency of Self-study (Ref.: Never) | | | | |
| Rarely | 0.219 | 1.252 | -0.0327 | 0.540 |
|  | (0.519) | (1.074) | (0.629) | (1.108) |
| Occasionally | 0.455 | 1.360 | 0.0407 | 0.724 |
|  | (0.497) | (1.048) | (0.597) | (1.062) |
| Sometimes | 0.994^*^ | 1.605 | 0.307 | 0.965 |
|  | (0.492) | (1.042) | (0.588) | (1.049) |
| Often | 1.704^***^ | 1.735 | 0.605 | 1.371 |
|  | (0.492) | (1.042) | (0.588) | (1.047) |
| Year (Ref.: 2011) |  |  |  |  |
| 2012 | -0.334^***^ | 0.197 | 0.128 | -0.0914 |
|  | (0.0869) | (0.130) | (0.110) | (0.164) |
| 2013 | 0.0914 | 0.621^***^ | 0.383^**^ | 0.545^***^ |
|  | (0.0973) | (0.132) | (0.117) | (0.156) |
| 2014 | 0.134 | 0.114 | -0.0221 | 0.323 |
|  | (0.102) | (0.153) | (0.131) | (0.172) |
| Constant | — | -3.474^**^ | -1.431^*^ | -3.757^***^ |
|  |  | (1.081) | (0.635) | (1.107) |
| Cutpoint 1 | -2.026^***^ |  |  |  |
|  | (0.530) |  |  |  |
| Cutpoint 2 | -0.0424 |  |  |  |
|  | (0.527) |  |  |  |
| Cutpoint 3 | 1.472^**^ |  |  |  |
|  | (0.527) |  |  |  |
| Cutpoint 4 | 3.193^***^ |  |  |  |
|  | (0.529) |  |  |  |
| *N* | 3011 | 2960 | 2974 | 2796 |
| pseudo *R*^2^ | 0.058 | 0.037 | 0.037 | 0.052 |

Standard errors in parentheses

^*^ *p* < 0.05, ^**^ *p* < 0.01, ^***^ *p* < 0.001
